# Coaches’ perceptions on qualities defining good adolescent rugby players and are important for player recruitment in talent identification programs: the SCRuM project

**DOI:** 10.1186/s13104-019-4170-y

**Published:** 2019-03-13

**Authors:** M. Chiwaridzo, N. Munambah, S. Oorschot, D. Magume, J. M. Dambi, G. Ferguson, B. C. M. Smits-Engelsman

**Affiliations:** 1 Division of Physiotherapy, Department of Health and Rehabilitation Sciences, Faculty of Health Sciences, University of Cape Town, Cape Town, South Africa; 2 Division of Occupational Therapy, Department of Health and Rehabilitation Sciences, Faculty of Health Sciences, University of Cape Town, Cape Town, South Africa; 3 Department of Rehabilitation, Academic Medical Center, University of Amsterdam, Amsterdam, Netherlands; 4 Rehabilitation Clinic, Premier Service Medical Investment, 36 Josiah Tongogara, Avenue, Harare, Zimbabwe; 5 Rehabilitation Department, College of Health Sciences, University of Zimbabwe, P.O Box, A178, Avondale, Harare Zimbabwe

**Keywords:** Rugby, Perceptions, Qualities, Skills, Attributes, Qualitative study, Coaches

## Abstract

**Objective:**

Competitive rugby is increasingly becoming popular among adolescent players even in countries hardly known for rugby such as Zimbabwe. Given the increased participation rates, burgeoning talent identification (TID) programs and the reportedly high injury-risk associated with competitive youth rugby, the minimal qualities or skills needed for effective performance by all young players need further clarification. Therefore, this qualitative study was conducted to explore the perceptions of high-school based rugby coaches on the key qualities or skills defining good adolescent rugby players and should be considered for player recruitment in TID programs. Currently, there is no consensus in literature from the coaches’ perspective on these qualities.

**Results:**

The final sample had 22 coaches (median age = 45.5 years) with years of coaching high-school rugby ranging from 6 to 17 years. Using the conventional approach to inductive content analysis four broad themes emerged suggesting the multifaceted nature of the requirements imperative for effective and optimal rugby performance among adolescent rugby players as perceived by the coaches. Themes identified included: physiological characteristics, anthropometric attributes, psychological qualities and game-specific skills. Possibly, training approaches or design of rugby-specific test-batteries should consider all these important qualities and be multi-dimensional in composition.

**Electronic supplementary material:**

The online version of this article (10.1186/s13104-019-4170-y) contains supplementary material, which is available to authorized users.

## Introduction

Globally, the number of adolescents playing competitive rugby is increasing even in countries where rugby is still developing such as Zimbabwe [1–3]. Due to high injury risk associated with rugby [4–7], adolescents playing competitive rugby should possess qualities needed for competition. Rugby is a physically challenging sport requiring specialist training of participants in preparation of the physiological demands and sporadic intensities [8, 9]. Hence, coaches should ensure that young players have qualities commensurate with rugby demands [10–12].

The need for talented young rugby players is a long-term vision for many countries [13]. Consequently, rugby bodies are constantly developing models for early identification of athletes [14–16]. Furthermore, demand for knowledge on rugby requirements continues to stimulate research on qualities discriminating players [17–19]. Previous studies have shown that anthropometric, physiological, rugby-specific skills and psychological attributes either determine team selection or discriminate rugby players by playing abilities [20–23], indirectly suggesting qualities important for rugby. However, little is known whether rugby coaches’ value these identified qualities or have different perceptions based on practical and contextual experience. Currently, literature is equivocal on the qualities coaches perceive important or should be emphasised for training [24]. In Zimbabwe where high-school rugby is still developing and mainly played by school boys, it is interesting to explore what local coaches perceive as the most important qualities characterising good adolescent rugby players and are important when recruiting young rugby talent. Such studies potentially inform composition of future test batteries designed for talent identification (TID) programmes. Therefore, the present study sought rugby coaches’ perceptions on qualities defining a good adolescent rugby player and are important for consideration in player recruitment in TID programs.

## Main text

### Study design, setting and participants

A qualitative study design was employed to determine qualities characterising good adolescent rugby players from coaches’ perspective. This study was part of a project called the School Clinical Rugby Measure (SCRuM) partly described elsewhere [25, 26]. The present study targeted coaches of high-school male adolescent rugby players in Zimbabwe and was conducted at a school hosting the 2017 Dairiboard Zimbabwe Schools Rugby Festival (DZSRF). The festival is an annual national youth rugby tournament featuring rugby-playing primary and secondary schools. Reportedly, 150 schools participated in the 7-day long tournament in 2017 [27]. The concept behind DZSRF is similar to the national youth rugby tournaments held in South Africa designed for U-13 to U-18 rugby players [28–31].

### Sampling and eligibility criteria

The coaches were selected with an *apriori* intention of maximum heterogeneity within the sample. This entailed purposively-recruiting coaches from schools playing in the three high-school rugby leagues. The leagues include the Super Eight Schools Rugby League (“elite league”), Co-educational School Rugby League (“sub-elite”) and the Interscholastic High Schools Rugby League (“amateur league”) [26]. Firstly, a list of all participating high schools was obtained from the DZSRL director. The schools were then stratified into three categories representing the domestic high-school rugby leagues. Out of the 42 coaches approached from various schools, 22 volunteered to participate. The participants’ demographic characteristics and rugby-related information are presented in Table 1. However, full-time rugby coaches with at least 5 years of Under-13 to Under-19 coaching experience were eligible. Additionally, coaches previously involved in TID programmes were purposively recruited for that unique experience.

**Table 1:** Sample demographic characteristic and rugby-related information (N = 22)

Characteristic	Response	n (%)
Type of school coached	Government	10 (45.5)
	Private	12 (54.5)
High-school rugby league the school plays in^a^	Super Eight Schools Rugby League (elite)	11 (50.0)
	Coeducational Schools Rugby League (sub-elite)	5 (22.7)
	Interscholastic Schools Rugby League (amateur)	6 (27.3)
Age of the participants (years)	30–39	3 (13.6)
	40–49	16 (72.8)
	50+	3 (13.6)
Median age (45.5 years)		
Interquartile range = 42–49		
Number of years coaching high school rugby in total (years)	≤ 9	16 (72.7)
	10+	6 (27.3)
School rugby team currently being coached	Under 13 s	1 (4.5)
	Under 15 s	2 (9.1)
	Under 16 s	5 (22.7)
	Second team	5 (22.7)
	First team	9 (40.9)
Number of years coaching current school rugby team (years)	1–2	7 (31.8)
	3–4	8 (36.4)
	5–6	3 (13.6)
	7–8	3 (13.6)
	9–10	1 (4.5)
Other rugby coaching experience	Yes	4 (18.2)
	No	18 (81.8)
Have played rugby in lifetime	Yes	22 (100)
	No	0 (0)
Years played rugby in total (years)	1–9	3 (13.6)
	10+	19 (86.4)

^a^Super Eight Schools Rugby League represents the best high school rugby league in the country featuring eight top rugby playing high schools in the country. The league is regarded as the “elite” league. Co-educational Schools Rugby League represents the second most competitive league in the country (sub-elite) and the Interscholastic Schools Rugby League represent high school rugby league for the rest of the schools playing rugby in the county mainly composed of amateur players

### Procedure

Ethical approval was granted by the Human Research Ethics Committee (HREC) of the University of Cape Town (reference: 016/2016). Participant identification and data collection were conducted between 30 April and 6 May, 2017, with at least two interviews scheduled per day. Coaches were directly approached by the first author seeking participation. A verbal explanation highlighting the rationale and study procedural issues was given. Potential participants were also left with information letters further detailing study purpose and methodology. Interview appointments would be set with the willing coaches, agreeing on the most convenient time and place for the interview.

On agreed dates, coaches would be invited to a quiet classroom specifically allocated for the interviews. Prior to the interviews, participants provided written informed consent and were verbally assured that the information provided was confidential. Coaches’ demographic and rugby-related information was elicited first. Subsequently, a trained research assistant conducted semi-structured face-to-face interviews in English since all participants understood and spoke the language. The interviews were based on a face-and-content validated English interview guide (Additional file 1) specifically developed and pre-tested for this study. Participants were probed for clarity and elaboration when necessary. All the interviews were audio-recorded and lasted between 15 to 40 min.

### Data analysis

For this article, we only analysed transcribed data from the second part of the interview guide. The interview data was transcribed verbatim by an independent person. Subsequently, the transcripts were proof-read by the first author against the original audio material verifying transcription accuracy. Noted discrepancies were discussed with the interviewer and transcriptor for a mutual consensus. Participant checking of the transcripts was then conducted with a convenience sample of rugby coaches (n = 12). Data analysis commenced immediately after member checking. The manifest and latent content of the transcriptions was analysed according to the conventional approach of inductive content analysis to generate categories and themes [32–34]. Conventional content analysis is commonly used to analyse textual data preferably when literature on a phenomenon is limited [32]. Literature documenting coaches’ perceptions on qualities defining good adolescent rugby players is sparse. Content analysis of textual data was manually conducted by the first author following steps of decontextualisation, recontextualisation and categorisation as described in literature [35]. A summary of the analytical process is provided in Additional file 2.

### Decontextualisation

Firstly, the first author read several times the entirety of transcribed data. This process of “immersion” was to gather a general understanding of the data [32, 34–36]. Then, each transcript was carefully read specifically highlighting sentences or phrases (*meaning units*) expressing qualities defining good rugby player. The highlighted meaning units were then “copy-pasted” onto a new document and further condensed into smaller meaning units, called *condensed meaning units* [34, 35]. The next step involved inductively creating an initial code list from the condensed meaning units of three randomly selected transcripts in a process called *open*-*coding* [32, 37]. This process was done to minimise change of the codes during the process of coding rest of the transcripts [35]. Using the generated code list as a guide, the first author then coded rest of the 19 transcripts and adding new codes when necessary [32].

### Recontextualisation and categorisation

After coding, the first author read several times the meaning units, condensed meaning units and emergent codes to confirm or re-code the condensed meaning units. Subsequently, the researcher “copy-pasted” all the emergent codes onto a Microsoft Excel document, comparing the codes for possible similarities and differences [36]. Similar codes were coalesced and different codes remained distinct. Codes were then sorted into sub-categories and furthermore, into categories for the manifest content. Finally, the underlying meaning of the categories representing the latent content of the data was examined generating themes [36]. Although coding was undertaken by the first author, investigators’ triangulation was conducted to validate the process. This was done by having the second (NM) and third authors (SO) code the transcriptions independently and derive own categories and themes. The authors (MC, SO, NM) had to compare the coding, sub-categorisation, and categorisation, discussing reasons for developed themes. Differences were solved by revisiting textual data, derived meaning units and condensed meaning units, and mutually re-coding the data, and agreeing on appropriate sub-categories, categories and themes.

## Results

The four major themes representing qualities defining good adolescent rugby players and are important for player recruitment in TID programs that emerged from the coaches perceptions were: (i) physiological characteristics, (ii) anthropometric attributes, (iii) game-specific skills, and (iv) psychological qualities (Table 2). Additional file 3 further depicts emergent codes and illustrative quotes from selected coaches in detail.

**Table 2:** Results of thematic analysis of coaches perceptions on qualities characterising good adolescent male rugby players (n = 22)

Theme	Category	Sub-category
Physiological characteristics	Muscular strength	Total body strength
		Upper-body muscular strength
		Lower-body muscular strength
	Muscular power	Total muscular power
		Upper-extremity power
		Lower-extremity
	Agility	Agility
	Endurance	Endurance
	Speed	Speed
		Repeated running
	Anaerobic capacity	Anaerobic capacity
	Balance	Balance
	Coordination	Coordination
	Muscle flexibility	Muscle flexibility
	Repeated effort ability	Repeated high intensity effort
Anthropometric variables	Physical qualities	Body mass
		Height
		Body composition
Game-specific skills	Basic technical skills	Passing
		Kicking
		Catching
		Tackling
		Evasion
		Ball handling skills
		Defensive/offensive skills
	Perceptual-cognitive skills	Auditory skills
		Visual skills
		Anticipatory skills
		Decision making
		Game sense
		Adaptability
	Miscellaneous	Communication skills
		Leadership
		Competitiveness
		Team player
Psychological qualities	Mental strength	Mental strength
	Emotional stability	Emotionally stable
	Attitude and personal traits	Positive attitude
		Courageous
		Determined
		Disciplined
		Teachable
		Passionate

## Discussion

The present study revealed ten categories under physiological qualities, confirming findings from previous studies that rugby requires possession of many physiological qualities [17, 24]. Muscular strength and power were perceived to be crucial in match situations involving aggressive contact, lifting, tackling, running and scrummaging. Rugby is a full-contact sport warranting optimal muscular strength and power [38–40]. There is compelling evidence that muscular strength and power discriminates rugby players for team selection [41–43]. Endurance and anaerobic capacity were also perceived to be important. The coaches felt that rugby players should show excellent recovery after repeated engagements in physical episodes. This is because rugby is an intermittent sport characterised by high-intensity anaerobic activities separated shortly by low-intensity aerobic activities and rest [12, 44, 45]. Elite junior rugby league players were previously found to have superior maximal aerobic capacity when compared to sub-elite players [46, 47]. Speed, aerobic capacity, muscular endurance, and body mass predicted players selected for junior rugby teams [23]. In the present study, coaches’ perceived speed as important and emphasised the importance of running ability. There is evidence correlating speed and repeated-sprint ability to game performance such as tackle breaks and tries scores [19]. Also, previous studies illustrated the importance of speed and agility [46, 47].

Anthropometric qualities such as appropriate height and optimal body mass were also perceived as important. In support, significant differences were found in body mass and composition between U-14 rugby players playing competitive rugby compared to those playing at a more recreational level [43]. Skin-fold thickness significantly contributed to the discriminant analysis of selected and non-selected players in professional rugby league [41]. In a study observing changes in body size and anthropometric characteristics of U-20 rugby players, Lombard et al. [48] found that players became heavier and taller over time. Also, Darrall-Jones et al. [20] demonstrated significant increases across age categories for body mass and height. These findings reflect the nature of the sport which emphasise appropriate anthropometrics attributes for rugby players [21, 22, 49, 50].

Coaches also perceived passing, catching, tackling and ball-handling skills as important. These findings were also shared by other authors [39, 51–53]. Additionally, the coaches perceived that perceptual-cognitive skills including decision-making ability, game-sense awareness, anticipation, visual and auditory skills were important. Hendricks [39] reported almost similar findings. Professional rugby league players had better tackling proficiency, pattern recall (ability to read the game) and prediction ability (decision-making), and reactive agility when compared to semi-professional players [54, 55]. Moreover, measures of technical and perceptual skills were associated with more line-breaks assists, try assists, and tackles completed during competition [56].

In the present study, psychological qualities were also perceived to be important although there are limited studies corroborating this evidence. Tredrea et al. [23] found no significant differences between selected and non-selected adolescent players for psychological attributes using the Mental Toughness (MT) Questionnaire. However, other studies involving adult players reported contrasting results using same tool [57]. Tredrea et al. [23] attributed that to reduced discriminative ability of MT tool in adolescents. Nevertheless, Larkin and O’Connor [58] found that perceived qualities such as “coachability” and “positive attitude” were important in soccer, an intermittent team sport like rugby. These findings were also shared by the coaches in the present study. The coaches felt that adolescent rugby players should be “teachable” and have personal attributes such as courage, determination, hardworking, discipline and passion for the sport.

In conclusion, coaches perceived physiological, anthropometric, and psychological and game-specific skills to be defining good adolescent rugby players. Therefore, training approaches or test-battery design should incorporate all these important qualities. Additionally, consistent with the training periodisation approach [59], coaches may utilise this information during the coaching process and in improving player performance.

## Study limitations

- Although generalisation of study findings is not important in qualitative studies, the 22 participants represented all the high-school rugby leagues in the country. Nevertheless, one major limitation was that data analysis was not conducted iteratively based on the saturation concept.
- Investigator triangulation of the coding process conducted provides credibility to the findings. However, credibility could have been enhanced by eliciting the perceptions of male adolescent rugby players’ playing competitive school rugby and further, ensuring that member checking of the transcripts involved not only a convenience sample of 12 rugby coaches as used in the present study.

## Additional files

**Additional file 1.** Interview guide for the qualitative study.

**Additional file 2.** Summary of the qualitative content analysis process from data transcription to formulation of themes.

**Additional file 3.** Emergent themes, sub-categories, categories and themes from the interview data.

## Abbreviations

DZSRL: Dairiboard Zimbabwe Schools Rugby Festival
HREC: Human Research Ethics Committee
MT: mental toughness
MRCZ: Medical Research Council of Zimbabwe
SCRuM: School Clinical Rugby Measure
TID: talent identification
U: under
