# Supplementary material for: Coaches’ perceptions on qualities defining good adolescent rugby players and are important for player recruitment in talent identification programs: the SCRuM project
Source: BMC Res Notes. 2019 Mar 13;12:132. doi: 10.1186/s13104-019-4170-y (PMC6417159; doi:10.1186/s13104-019-4170-y)
Supplement: Supplementary file 1 — Additional file 1. Interview guide for the qualitative study. [file 13104_2019_4170_MOESM1_ESM.docx]

Participant Reference Number…………… Date………..…………

**Demographic and school-related information**

1. How old are you? (Full years attained):
2. What is the name of your school?
3. What type of school is it?
4. In which high-school rugby league does your school participate?

**Rugby-coaching related information**

1. Overall, how many years in total have you been a high-school rugby head coach?
2. When did you start coaching rugby at this particular school as a head coach?
3. Which school team(s) are you currently coaching at the present moment at the school?
4. For how long have you been coaching this current school team(s)?
5. Which other rugby school team(s) have you been previously involved with either as a head coach or assistant coach at this school before?
6. Do you have any other rugby coaching experience besides in high schools?
7. If yes, please specify where else you have coached previously

**Personal rugby experience**

1. Have you ever played rugby in your lifetime?
2. For how many years in total did you play rugby?
3. At what level did you play rugby?

**The Interview Guide**

**First part: Source of rugby players and criteria for player selection**

1. What do you think motivates high school boys to take up rugby as a sport in school?
2. Where do the young adolescent players who get to play high-school rugby at your school come from?
3. Who selects the players to be included in the school rugby teams at each playing level from the Under 13 to Under 19?
4. What criteria is used for the selection of players into a school rugby team?

**Second part: Qualities important in rugby**

1. What individual qualites, attributes, or skills do you think makes a good adolescent rugby player?
2. Given an opportunity to participate in talent identification and recruitment programme in Zimbabwe, what individual qualities, attributes or skills would you consider or look for among young potential players?
3. Give me one example of a rugby player in your school team you consider exceptionally good in playing rugby, explaining why you think he is such a good young player in terms of the qualities, attributes or skills he possesses?

**Third part: Methods of assessing identified qualities**

1. For each quality, attribute or skill you identified to be defining a good adolescent rugby player and important to conisder for player recruitment, what test(s) or methods of assessment do you frequently use to assess for those qualities among your players?
