# Supplementary material for: Coaches’ perceptions on qualities defining good adolescent rugby players and are important for player recruitment in talent identification programs: the SCRuM project
Source: BMC Res Notes. 2019 Mar 13;12:132. doi: 10.1186/s13104-019-4170-y (PMC6417159; doi:10.1186/s13104-019-4170-y)
Supplement: Supplementary file 2 — Additional file 2. Summary of the qualitative content analysis process from data transcription to formulation of themes. [file 13104_2019_4170_MOESM2_ESM.docx]

Sub-categorisation, categorisation and theme formulation

Themes

Open coding-generating initial coding list inductively

Condensing meaning units of analysis

Confirmation and re-coding the condensed meaning units

Grouping-comparing codes for similarities and differences

Recontextualisation and categorisation

Decontextualisation

Selecting meaning units of analysis

Reading transcribed data several times “immersion”

Data coding based on inductive coding list

Figure S1: Summary of the content analysis process
