# Supplementary material for: Coaches’ perceptions on qualities defining good adolescent rugby players and are important for player recruitment in talent identification programs: the SCRuM project
Source: BMC Res Notes. 2019 Mar 13;12:132. doi: 10.1186/s13104-019-4170-y (PMC6417159; doi:10.1186/s13104-019-4170-y)
Supplement: Supplementary file 3 — Additional file 3. Emergent themes, sub-categories, categories and themes from the interview data. [file 13104_2019_4170_MOESM3_ESM.docx]

**Additional file 3:** Emergent themes, categories, sub categories and codes from interview data with selected illustrative quotes from coaches

| **Theme** | **Category** | **Sub-category** | ***Emergent Codes** | **Selected illustrative comments from coaches** |
| --- | --- | --- | --- | --- |
| Physiological characteristics | Muscular strength | Total body strength | Strong, strong body, strength, sturdy | *“..rugby is a man’s game you need to be strong, it involves a lot of contact, which is aggressive and requires players that are able to resist the pushes, the pulls and tackles. You need to be strong”* ***(Participant MF01)*** |
|  |  | Upper-body muscular strength | Forearm, arm, hand, neck, shoulder, and trunk strength | *“So you need strength in your neck muscles, shoulders, forearms, arms and hands and wrists. Shoulder strength helps [...] with high and low tackles, […] forearm and arm strength plus trunk enables you to push hard through defensive walls, hands and wrists strength holds the ball tight whilst running, in catching and in passing...”* ***(Participant AW01)*** |
|  |  |  |  |  |
|  |  | Lower-body muscular strength | Lower leg strength, thigh strength | *“Thigh strength gives you speed to push through, in scrums it helps forward players scrummage. Lower leg strength is important for running and I think especially for adolescents because their rugby is mainly running a lot they need lower strength, and power, strength in these muscles and in the calf muscles as well for your sprints”* ***(Participant AW01)*** |
|  |  |  |  |  |
|  | Muscular power | Total muscular power | Power, powerful | *“Forwards spend a lot of time wrestling for the ball, they get into tackles, so they need balance, strength and power and good vision and good communication skills with each other.”* ***(Participant DZ01)*** |
|  |  |  |  |  |
|  |  | Upper-extremity power | Arm-power | *“I think you need strength, and arm power to play through defenders whilst running, holding the ball but they must also be lean enough to carry their body, to run…”* ***(Participant HE01)*** |
|  |  |  |  |  |
|  |  | Lower-extremity power | Leg power | *“Rugby is physical from the U13s to the U19s it’s about power to make tackles, resisting tackles, all the aggression, power in sprinting, power in our legs during lineout and when going for kicked balls”(****Participant STJ01)*** |
|  |  |  |  |  |
|  | Agility | Agility | Change of direction, agility | *“…agility as well is important in all players especially for wingers, flanks, and eighth man. Agile players often evade defenders smoothly and run down the defensive line and make a break. Because in rugby […] you can score from a wide position […], so change of direction is crucial.”* ***(Participant DZ01)*** |
|  |  |  |  |  |
|  | Endurance | Endurance | Endurance, aerobic capacity, indefatigable, stamina | *“… young players need endurance, it is very important. I would say the number one quality is endurance, because you can imagine form one students Under-13 players running around, for 40 minutes or form fours your Under-17 running for 70 minutes with only a 10minutes break.”* ***(Participant AW01)*** |
|  |  |  |  |  |
|  |  |  |  | *“You know if you are not fit, physically fit, it takes about 5 minutes and you are tired already, you cannot run anymore and your legs are getting cramps and you can’t continue, juts 5minutes only and you are done, so you need to be fit, you need to be very healthy, and show that you have aerobic and anaerobic capacity”* ***(Participant STJ01)*** |
|  |  |  |  |  |
|  | Speed | Speed | Ability to accelerate, run, running ability, fast, good speed , sprinting | *“I also look at speed, for all players. Although there are certain positions which require speed than others but every rugby player must demonstrate the ability to run, accelerate when need arises, when in situations of attacking and defending.”* ***(Participant AW01)*** |
|  |  |  |  |  |
|  |  |  |  | *“If you don’t have the speed, and the strength and the ability to tackle well, then you are doomed. The opponents will beat you”* ***(Participant F01)*** |
|  |  |  |  |  |
|  |  | Repeated running | Keep running, continued running, speed endurance, repeated running | *“So speed is important and young players should demonstrate ability to run over and over several distances whether short or long without getting tired for them to enjoy rugby*.” **(*Participant DZ01)*** |
|  |  |  |  |  |
|  |  |  |  | *“Good players ought to have speed and show that they can run without easily getting tired. There is less time to rest in rugby but most of the time its speedy running, its tackles, and the hard stuff.” (****Participant CBC01****)* |
|  |  |  |  |  |
|  | Anaerobic capacity | Anaerobic capacity | Anaerobic capacity, recovers well, recovers from high intense activities | *“…that ability to recover from the high intense activities and be able to quickly rest and quickly resume working under pressure in tackling, scrums, rucks and maul and to work when tired is crucial in rugby.” (****Participant STJ01)*** |
|  |  |  |  |  |
|  |  |  |  | *“Good rugby players at all level must have exceptional anaerobic capacity should be able to participate in high intensity activities continuously without showing signs of fatigue and so do that effectively”* ***(Participant PE02)*** |
|  |  |  |  |  |
|  | Balance | Balance | Balance, stay on feet | *“And also they need to have strength as well, you need to have muscle, to be powerful because you get involved in collisions and you fall to the ground you get tackled and so forth so you need to have stamina, that strength and balance so that you don’t just fall”* ***(Participant C01)*** |
|  |  |  |  |  |
|  |  |  |  |  |
|  | Coordination | Coordination | Hand coordination | *“So with speed, comes power, comes agility, comes flexibility and coordination as well, so you need all those qualities put together*. *Those are the major ones. I think”* ***(Participant ER01)*** |
|  |  |  |  |  |
|  | Muscle flexibility | Muscle flexibility | Flexibility | *“Yes, you also need muscle flexibility, balance, as well as coordination, endurance, speed, you should be able to accelerate and also decelerate and also be able to change direction without losing your balance.”* ***(Participant LOM01)*** |
|  | Repeated effort ability | Repeated high intensity effort | Repeated engagement in physical battles, scrummaging, running, tackling | *Rugby is about continuous engagement in physical battles, your scrum, your ruck, during mauls, it’s about fighting for the ball, sprinting with it, with little time to rest or recover fully so if you are a forward player so you need to be attentive.”* ***(Participant HE02)*** |
|  |  |  |  |  |
|  |  |  |  | *“Rugby is about running and you need to have the ability to continuously perform the physically challenging tasks such as running, tackling, scrummaging, for you to play rugby.”* ***(Participant ER02)*** |
|  |  |  |  |  |
| Anthropometric variables | Physical qualities | Body mass | Appropriate body mass, optimal mass, body weight | *“Every good player should be able to pass the ball […] and rugby players also need to have strength and power, appropriate height, and body mass which depends on the position they play.”* ***(Participant C02)*** |
|  |  |  |  |  |
|  |  | Height | Appropriate height, stature, tall height, short height | *“…also good players must have the height, body mass which is needed in the sport. Size is important for proper positioning of the player. […] a good player uses the physical qualities he has to the advantage of the team…”* ***(Participant LOM01)*** |
|  |  |  |  |  |
|  |  | Body composition | Lean mass , muscular, muscle, optimal body | *“You also need players who have a good balance of muscle and fat, not too much fat not excessively thin rugby players but players with optimal body composition especially considering they position they fancy playing”* ***(Participant STG02)*** |
|  |  |  |  |  |
| Game-specific skills | Basic technical skills | Passing | Accurate passing, good passing, passing credibility, purposeful passing | *You see players that make it to the highest level like myself have good skills, skills such as offensive skills, defensive skills, evasion skills, perceptual skills, being in the game the entire 70 minutes, good auditory and visual skills, good passing skills this is important for every position”* ***(Participant ES01)*** |
|  |  |  |  |  |
|  |  |  |  | *“In addition, skills are important, rugby is a technical sport so players who can pass the ball accurately, pass at the right time […] are good players”* ***(Participant AW01)*** |
|  |  |  |  |  |
|  |  | Kicking | Good kicker, kick, kicking for distance | *“To me, it’s not about the size of the players, their appearance, no; it’s about the skills, ball distribution skills, ball control after catching the ball, kicking and passing, tackling as well.”* ***(Participant STG01)*** |
|  |  |  |  |  |
|  |  |  |  | *“Where you need strength have players that are strong, where you need speed have players that have speed, but all rugby players must have the core skills of passing, catching, and kicking the ball…”****(Participant C01)*** |
|  |  | Catching | Catch, catching skills | *“Another skill that you need to be called a good player is catching. How you hold your hands to receive the pass, and then that ability to catch it is important. You begin to appreciate the importance of catching when your team is running to score a try and then someone miscatches a good pass, and the ball is taken away...”* ***(Participant F01)*** |
|  |  | Tackling | Effective tackles, good tackler, tackle correctly, tackling | *“Others include, good and effective tackle technique is important, all players need that, rugby is about bringing your opponents down and that must be done safely and to stop the ball from going forward.”* ***(Participant PE01)*** |
|  |  |  |  |  |
|  |  |  |  | *“But I would want all my players to have skills which are specific to rugby, because all rugby players have to kick the ball, have to pass, they should tackle and they should catch, they should control the ball, you know.”* ***(Participant STG01)*** |
|  |  |  |  |  |
|  |  | Evasion | Beat defenders, evasion skills, evade opponents, side stepping ability | *“I would also want kids with skills, skills are becoming very important in rugby, and skills are a like the cherry on top of the ice cream, you need skills to evade defenders…”* ***(Participant HE02)*** |
|  |  |  |  |  |
|  |  |  |  | *“If you look at U13 or U14, you don’t emphasise on acquiring strength, muscles and power, their rugby is different when compared to the U18 […] which is very physical or competitive, for the youngest players all what they really need is to be physically fit and have more of the technical skills for example, the kicking, the passing, evasion skills…”* ***(Participant P01)*** |
|  |  |  |  |  |
|  |  | Ball handling skills | Ball carrying, handling, shielding | *“But of course, these are higher skills, but all rugby players should have basic skills first such as passing, catching, kicking, tackling, running, and carry the ball.”* ***(Participant MF01)*** |
|  |  |  |  |  |
|  |  |  |  | *“…if we are at the grassroots level let’s say we are in Binga there, your lower your standard a bit, you just want kids who can display the core rugby skills, can they pass, can handle the ball, can they receive a pass, very general rugby skills because you are considering that this a resource-constrained environment.”* ***(Participant ES02)*** |
|  |  |  |  |  |
|  |  |  |  | *“I think you also need very good passing skills, passing while running and very vision and quick thinking, as you run, and also good catching of the ball, shielding it whilst running…”* ***(Participant HE01)*** |
|  |  |  |  |  |
|  |  | Offensive and defensive skills | Defensive abilities, offensive skills | *“Good rugby players are excellent in preventing scoring of tries, they should have good defensive and offensive abilities and all rugby players should be able to defend not just the backline players even the forward players they initiate the first team defence through offense so should be good with their defensive and offense play.”* ***(Participant C02)*** |
|  |  |  |  |  |
|  | Perceptual-cognitive skills | Auditory skills | Auditory perception, auditory skills | *“You see players that make it to the highest level like myself have good skills, skills such as offensive skills, defensive skills, evasion skills, perceptual skills, being in the game the entire 70 minutes, good auditory and visual skills...”* ***(Participant ES01)*.** |
|  |  |  |  |  |
|  |  | Visual skills | Good vision, visual acuity, watching the ball, vision | *“This is important, and for first team and second team players we emphasise during training the importance of vision, visual acuity and quickness in reacting to situations.”* ***(Participant LOM01)*** |
|  |  |  |  |  |
|  |  | Anticipatory skills | Anticipation, read intention of others, quick reaction to situation, | *“….we also wants players with very good anticipation […]. Players able to watch and read the game, see what is happening, and can anticipate what the opponent player wants to do* ***….”(Participant P01)*** |
|  |  |  |  | *“I would choose one the guys who plays prop, MD an upper six student, he has been playing prop since U13, and he has grown into a mature and complete player with the prerequisites, he is an exceptional player with very good vision, strength and good anticipation.”* ***(Participant HE01)*** |
|  |  | Decision making | Decision making, thinking fast | *“Like strength is important, so as good side-stepping, evasion skills, tackling, running and passing, you also on top of that want a player with good decision making and someone willing to play with others”* ***(Participant HE02)*** |
|  |  |  |  |  |
|  |  |  |  | *“Players who are aware of the next player around them, whether it’s his player mate or not, and makes good decisions with the ball.”* ***(Participant AW01)*** |
|  |  | Game sense | Alertness, reading game, understanding game, game sense, game awareness | *“I think what makes a good adolescent rugby player is not just passing and kicking, it’s how you evade defenders, the tries you convert […], how defensive you are, how you read the game and the ability to convert a loss to win through your individual skills.”* ***(Participant MF01)*** |
|  |  |  |  |  |
|  |  |  |  | *“Also knowing where to position yourself in important, a good player should always have at the back of their mind where the next person, who is the next person close to them, whether it’s their player or opponent. This is important, this is understanding the game.* ***(Participant PE01)*** |
|  |  | Adaptability | Adapt to game situations, adapts quickly | *“….you know it is an advantage to have a player who adapts to the game and can understand how various positions are played…..”* ***(Participant CBC01)*** |
|  | Miscellaneous | Communication | Communication skills | *“Rugby is about skills, so rugby players should be able to pass the ball, kick the ball, to tackle, to side step, to catch the ball, aim at a target and, also in all that rugby players should be able to communicate their body language their intentions and be able to read the intentions of others as well.”* ***(Participant C01)*** |
|  |  |  |  |  |
|  |  | Leadership | Command respect, directs play, encourages team | *“The reason why i think he is exceptional is because he plays his position very well, and he is a tactical player with a lot of strength and power. He commands respects from the boys and he is very good leader for the team during play…”* ***(Participant C01)*** |
|  |  | Competitiveness | Competitive, fight for possession, fight off tackles | *“One example would be MD; he plays for seconds and subs for the first team. His is the eight men for the team. He has good speed, and he works very hard, he fights for every ball and he does not stop fighting.”* ***(Participant HE02)*** |
|  |  |  |  |  |
|  |  | Team player | Belief in team work, cohesion, team work, team player | *“But we would look at team work, ability to work in a team…”* ***(Participant HS01)*** |
|  |  |  |  |  |
| Psychological qualities | Mental strength | Mental strength | Mental strength | *“We look at mental strength, rugby it’s a mental game and you need to have mental fortitude to go through the game….”* ***(Participant MF01).*** |
|  |  |  |  |  |
|  | Emotional stability | Emotionally stable | Emotionally stable | *“ .... You need to be emotionally stable, physically fit and have the skills to play as a good player…...”* ***(Participant ES01).*** |
|  |  |  |  |  |
|  | Attitude and personal traits | Positive attitude | Mentality to win, right attitude, positive attitude | *“I would want players that endure [….] players with the mentality to win [….] and who work hard not to lose.”****(Participant ES01).*** |
|  |  |  |  |  |
|  |  | Courageous | Courage , courageous, bold, confident, | *“That’s why am saying be courageous, dont be afraid to play with bigger players….”* ***(Participant ES02).*** |
|  |  |  |  |  |
|  |  | Determined | Determination, hard work, commitment, focussed, focus, motivated | *“You see it through hard work, determination […] if you are rugby player it should reflect on the pitch and even outside….”* ***(Participant P01)*** |
|  |  |  |  |  |
|  |  | Disciplined | Discipline, well-behaved | *“…..the right behaviour is important. You cannot mix rugby with girls you know, so you have to be [….] and well-disciplined to play rugby.”* ***(Participant PE03).*** |
|  |  |  |  |  |
|  |  | Teachable | Easy to teach, listens, follows instructions | *“Good rugby players’ listens to the instructions of their coaches on and off the pitch, they are teachable and are able to receive the instructions non-judgemental and unconditional and apply it in training and in competitive matches. This behaviour is important”****(Participant AW01)*** |
|  |  |  |  |  |
|  |  | Passionate | Heart for the game, interest, love, passion | *“Of course, I also consider things like do you have passion for the sport, rugby requires passion and love for the game. You may get injured for life so you have to love the game if you to excel and become a good player. Its love that makes you train hard and play hard as well”* ***(Participant CBC01)*** |

*Reflects all the codes that emerged from the interview data that were then sub-categorised and then categorised to form common themes. For example, for the theme of physiological characteristics under the category of muscular strength a number of codes emerged such as strong, strong body, strength and sturdy which were then coalesced and sub-categorised under total body strength.
